# Influences of Maternal Nutrition and Lifestyle Factors on Early Childhood Oral Health: A Systematic Review of Mechanisms and Intervention Strategies

**DOI:** 10.3390/children11091107

**Published:** 2024-09-10

**Authors:** Murad Alrashdi

**Affiliations:** Department of Orthodontic and Pediatric Dentistry, College of Dentistry, Qassim University, Burayadh 52571, Saudi Arabia; mu.alrashidi@qu.edu.sa

**Keywords:** maternal nutrition, early childhood oral health, vitamin D, maternal smoking, dental caries prevention

## Abstract

Background: This systematic review and meta-analysis examined the impact of maternal nutrition and lifestyle factors on early childhood oral health. The review focused on the effects of maternal vitamin D levels and smoking during pregnancy on children’s dental health outcomes. Methods: A comprehensive literature search was conducted across PubMed, Scopus, and Web of Science, yielding 23 that were included for analysis. The quality of the studies was assessed using the Newcastle–Ottawa Scale. The effect estimates were pooled through a random effect model. All analyses were carried out using the R program. Results: Most studies in our systematic review showed a significant association between maternal vitamin D and smoking during pregnancy and childhood dental health outcomes. Meta-analysis revealed a significant association between maternal vitamin D levels and children’s dental health (OR = 1.30, 95% CI: 0.49 to 3.45, *p* < 0.001). Maternal smoking during pregnancy was strongly linked to an increased risk of childhood dental caries (OR = 0.3290, 95% CI: 0.2089–0.4491, *p* < 0.0001). Conclusions: These findings underscore the crucial role of maternal health behaviors in shaping children’s oral health trajectories. This study emphasizes the need for integrated public health interventions promoting healthier maternal behaviors and early preventive dental care.

## 1. Introduction

Oral health is a critical component of overall health and well-being, starting from early childhood. The World Health Organization emphasizes the importance of oral health because it is integral to general health and quality of life, impacting essential functions such as eating, speaking, and social interaction [1]. Early childhood, especially the time from pregnancy through the early years of life, is particularly crucial for setting the foundation of good oral health [2]. During this period, the health behaviors and environmental influences determined by family practices play a pivotal role in shaping a child’s future oral health outcomes [1]. Research indicates that maternal health behaviors, including dietary habits and oral hygiene, significantly influence the development and health of the child’s primary teeth [3]. For instance, maternal intake of certain nutrients during pregnancy, such as calcium, vitamin D, and phosphorous, is directly associated with the calcification and health of the child’s dentition [4]. Furthermore, habits such as smoking and alcohol consumption during pregnancy have been linked to negative outcomes in oral health in children, such as the development of enamel hypoplasia and an increased risk for caries. The role of family health extends beyond genetics and prenatal care. The early introduction to oral hygiene practices within the family setting also determines the establishment of habits that can prevent dental diseases. Studies have shown that children whose families maintain regular dental care practices, including routine brushing and dental visits, are more likely to develop these habits themselves, leading to better oral health outcomes [5]. Additionally, family dietary habits play a significant role in the prevalence of dental caries in children. Diets high in sugars and carbohydrates are well documented to contribute to the higher incidence of caries, whereas balanced nutrition supports not only general but also oral health [6]. Despite the well-documented influence of these factors, there remains a significant gap in systematic reviews that focus on the comprehensive mechanisms through which maternal nutrition and lifestyle factors impact the oral health of their children. Moreover, while individual studies have explored aspects such as the impact of specific nutrients or the role of hygiene practices, there is a need for a broader synthesis that also considers intervention strategies that can be effectively implemented. The aim of this systematic review is to thoroughly examine the existing literature on the influences of maternal nutrition and lifestyle factors on the oral health outcomes of early childhood. This review seeks to identify and synthesize the underlying mechanisms by which these factors affect children’s oral health and to evaluate the effectiveness of current intervention strategies aimed at mitigating these effects. By consolidating this evidence, this study will contribute to the development of more targeted and effective public health initiatives and educational programs to improve early childhood oral health outcomes. Through a comprehensive understanding of these dynamics, the review intends to foster better policy-making and community health strategies that are rooted in evidence-based research.

## 2. Materials and Methods

This systematic review was conducted based on the Preferred Reporting Items for Systematic Reviews and Meta-Analysis (PRISMA).

### 2.1. Search Strategy

The literature search for this systematic review was conducted using PubMed, Scopus, and Web of Science to identify studies that report on the impact of maternal nutrition and lifestyle factors on the oral health of children. The following search was performed in Scopus and Web of Science: (“Maternal Nutrition” OR “Maternal Vitamin D intake” OR “Maternal Calcium intake” OR “Maternal Factors” OR “Maternal Smoking” OR “Maternal Obesity” OR “Maternal Alcohol Intake” OR “Maternal Lifestyle”) AND (“Oral Health” OR “Dental Caries” OR “Early Childhood Oral Health” OR “Pediatric Dental Health” OR “Early Childhood Caries” OR “Enamel Hypoplasia”). The following search was performed in PubMed: (“Maternal Nutrition” OR “Maternal Nutritional physiological phenomena” [MeSH] OR “Maternal Vitamin D intake” OR “Maternal Calcium intake” OR “maternal factors” OR “Smoking” [MeSH] AND “Pregnancy” [MeSH] OR “Obesity” [MeSH] AND “Pregnancy” [MeSH] OR “Maternal Alcohol Intake” OR “Maternal Lifestyle”) AND (“Oral Health” OR “Dental Caries” OR “Early Childhood Oral Health” OR “Pediatric Dental Health” OR “Early Childhood Caries” OR “Enamel Hypoplasia”). The search was limited to articles published in English from January 2000 until the present to ensure that the review reflects current knowledge and practices. A complete search strategy for each database is provided in Supplementary Material Tables S1–S3.

### 2.2. Study Selection

Specific selection parameters were established. Eligible studies included those with cross-sectional or longitudinal designs that examined the relationship between maternal nutrition or other lifestyle factors during pregnancy and the occurrence of dental caries in early childhood. Publications were required to present original data or provide statistical measures such as odds ratios, risk ratios, or hazard ratios, along with their 95% confidence intervals. Certain types of publications were deemed ineligible, including narrative reviews, case reports, correspondence, commentaries, animal experiments, laboratory studies, and conference summaries. Additionally, research that did not specifically address maternal nutrition or lifestyle factors on childhood dental caries as outcomes was excluded. Studies lacking comprehensive data or published in languages other than English were also omitted. Two reviewers independently screened titles and abstracts for eligibility. Discrepancies were resolved through discussion or by consulting a third reviewer if necessary. Eligible studies underwent full-text review to confirm inclusion.

### 2.3. Data Extraction

Data extraction from the included studies was conducted using a standardized form by two independent reviewers. The process captured key information such as study characteristics (including author names, publication year, study design, and sample size), specifics of the maternal factors under investigation (encompassing nutritional and lifestyle elements, as well as their timing during pregnancy or early childhood), and the oral health outcomes measured in children. Additionally, the extraction focused on the principal findings that elucidate the influence of maternal factors on children’s oral health. Any disagreements arising during the data extraction process were resolved through discussion between the reviewers or, if necessary, through arbitration by a third party. This systematic approach ensured a comprehensive and unbiased collection of relevant data from the selected studies.

### 2.4. Quality Assessment

The Newcastle–Ottawa Scale (NOS) will be employed to evaluate the quality of the non-randomized studies included in this meta-analysis. This assessment tool examines studies from three primary perspectives: the selection process for study groups, the comparability between these groups, and the method of ascertaining either the exposure (in case–control studies) or the outcome of interest (in cohort studies). By utilizing this standardized approach, we aimed to provide a comprehensive and objective assessment of each study’s methodological rigor and potential for bias. This quality assessment was crucial for interpreting the reliability and validity of the findings from individual studies and their overall contribution to the meta-analysis.

### 2.5. Data Synthesis and Analysis

In this meta-analysis, we utilized odds ratios (OR) with 95% confidence intervals (CI) for cross-sectional studies and risk ratios (RR) with 95% CI for longitudinal studies to present our findings. To assess heterogeneity among the included studies, we employed I^2^ statistics and the Q test, with low heterogeneity defined as *p* > 0.1 or I^2^ < 50%. The selection of the pooling model was contingent upon the level of heterogeneity: a random-effects model was chosen for significant heterogeneity, while a fixed-effects model was used when heterogeneity was low. To evaluate potential publication bias, we implemented funnel plots. All statistical analyses were performed using R (version 4.3.1; R Core Team, Vienna, Austria, 2023). 

### 2.6. Risk of Bias Assessment

Potential publication bias in the studies included in the meta-analysis was assessed by plotting the standard error of the log odds ratio against the corresponding log odds ratio in a funnel plot. The symmetry of the funnel plot was then visually examined to assess for any asymmetry. Additionally, both Begg’s test and Egger’s regression were conducted to provide statistical evaluations of publication bias where appropriate. A *p*-value < 0.05 from either of these tests suggested the presence of significant publication bias in the included studies.

### 2.7. Ethical Considerations

As this study was a systematic review, it compiled data from previously published research and did not involve direct interaction with human or animal subjects. Consequently, ethical approval was not necessitated for this analysis. Nevertheless, the review was conducted in strict adherence to the ethical standards established by the 1964 Declaration of Helsinki and its subsequent amendments.

## 3. Results

### 3.1. Study Selection

**Figure 1:**
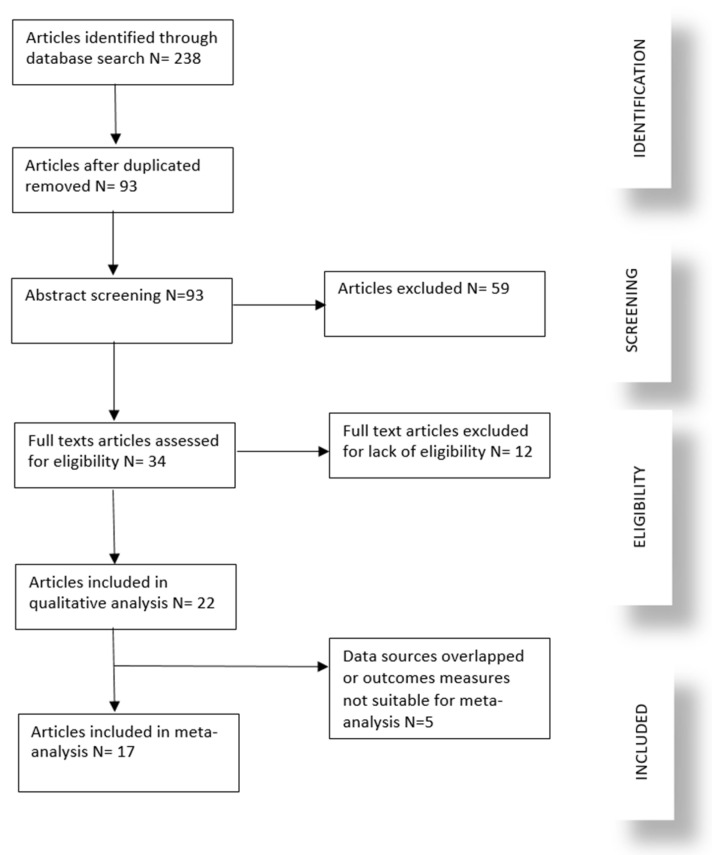
Flow chart for literature search process.

The initial search yielded a total of 238 articles from PubMed, Scopus, and Web of Science. After duplicates were removed, 93 articles remained. Screening titles and abstracts excluded 59 articles that did not meet the inclusion criteria, primarily due to irrelevance to the maternal factors or child oral health outcomes. The full texts of the remaining 34 articles were assessed for eligibility, resulting in 22 studies being included in the qualitative analysis. Studies (12) were excluded from the qualitative analysis based on our predefined inclusion and exclusion criteria. Of these, 11 studies did not specifically address the relationship between maternal nutrition or lifestyle factors and childhood dental caries outcomes. One study was published in a language other than English, preventing its inclusion in our review. Subsequently, five studies were excluded from quantitative synthesis (meta-analysis) because the outcome measures were not suitable for meta-analysis (3) and data sources overlapped (2). The process of study selection is summarized in a PRISMA flow diagram, as shown in Figure 1.

### 3.2. Study Characteristics and Outcomes

The 22 included studies varied in terms of design, population, and geographic location but predominantly consisted of cohort studies (*n* = 11), followed by cross-sectional (*n* = 8) and randomized trials (*n* = 3). These studies were conducted across several countries, with the majority from countries in Europe and America. Studies showed a mixed but generally concerning association between maternal vitamin D status and dental outcomes in children. For instance, vitamin D insufficiency during pregnancy was linked to increased risks of dental caries and enamel defects, with some studies reporting significant associations between low maternal vitamin D and developmental enamel defects. Notably, high-dose vitamin D supplementation showed protective effects against enamel defects in one study [7]. However, other studies found no significant associations, suggesting variability in outcomes across different populations and study designs. For instance, Nørrisgaard et al. [7] conducted a randomized trial with a substantial sample of 623 mothers and 588 children, finding that high-dose vitamin D supplementation was associated with a reduced risk of enamel defects, with odds ratios (OR) of 0.47 and 0.50 for permanent and deciduous dentitions, respectively. Similarly, Beckett et al. [8] had a smaller sample of 81 participants but reported a strong association between third-trimester vitamin D insufficiency and increased risk of caries by age 6, with an incidence rate ratio (IRR) of 3.55. Studies like Van der Tas et al. [9] and Mortensen et al. [10] involved larger samples of 4750 mothers and 1241 participants, respectively, but did not find significant associations between vitamin D levels and hypomineralized second primary molars (HSPM) or molar incisor hypomineralization (MIH). Studies on maternal smoking and childhood carries had sample sizes ranging from 1687 to 76,920 participants. They consistently report a positive association between maternal smoking and childhood dental caries. Although the majority of studies show a heightened risk, the strength of the association varies, with some studies reporting stronger correlations and others showing more modest effects. A detailed summary of this study characteristics and outcomes is provided in Table 1 and Table 2, which include authors, year of publication, study design, sample size, and key findings.

### 3.3. Quality of Included Studies

The Newcastle–Ottawa Scale (NOS) scores ranged from 6 to 9 for cohort and randomized trial studies and 6 to 8 for cross-sectional studies. The majority of studies scored between 7 and 8, indicating generally good methodological quality. Randomized trials, such as those by Børsting et al. (2022) and Nørrisgaard et al. (2019), received the highest scores of 9/9, reflecting their strong design and execution. Prospective cohort studies scored slightly higher than retrospective cohorts, achieving 7–8/9. Cross-sectional studies generally received the lowest scores, ranging from 6/9 to 7/9. The most common areas where studies lost points were in the selection and comparability domains, while most studies scored well in the outcome assessment domain. Overall, the NOS scores suggest that the majority of the studies included in this analysis were of high methodological quality, with some variation based on study design and specific methodological strengths and limitations.

### 3.4. Meta-Analysis

#### 3.4.1. Maternal Vitamin D Intake Is Associated with Reduced Early Childhood Carries and Enamel Defects

**Figure 2:**
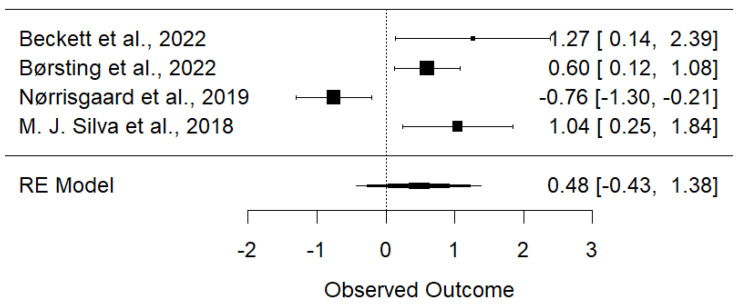
Forest plot of the association between maternal vitamin D and children’s dental health [7,8,11,14].

**Figure 3:**
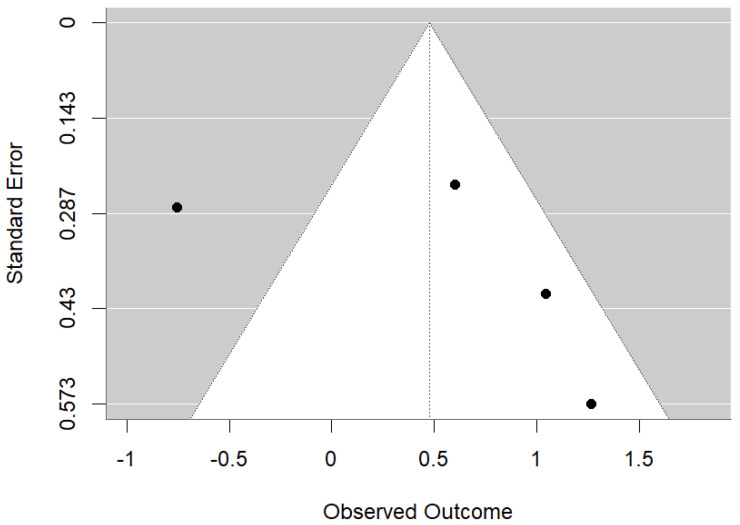
Funnel plot assessing publication bias in studies of maternal vitamin D and children’s dental health.

The meta-analysis evaluated the association between maternal vitamin D levels and dental health outcomes in offspring, revealing a significant overall association (OR = 1.30, 95% CI: 0.49 to 3.45, *p* < 0.001) (Figure 2). The analysis showed substantial heterogeneity (Q = 21.815, I^2^ = 86.2%, *p* < 0.001), indicating variability among the studies. Separate analysis of cohort studies found a statistically significant overall effect (OR = 1.1187, 95% CI: 0.4680–1.7694 *p* < 0.001). There was no observed heterogeneity among the cohort studies (I^2^ = 0.00%), as confirmed by a non-significant Q test = 0.1007, *p* = 0.7510. This suggests that the effect is consistent across the studies and the observed effect is robust. Randomized trials supported the beneficial effect of vitamin D supplementation during pregnancy on reducing enamel defects. However, the high heterogeneity suggests diverse results, likely due to differences in study designs, populations, and methods. The funnel plot (Figure 3) suggests some potential asymmetry in the data, but the small number of studies limited the strength of any conclusions about publication bias.

#### 3.4.2. Maternal Smoking Is Associated with Increased Risk of Early Childhood Carries

**Figure 4:**
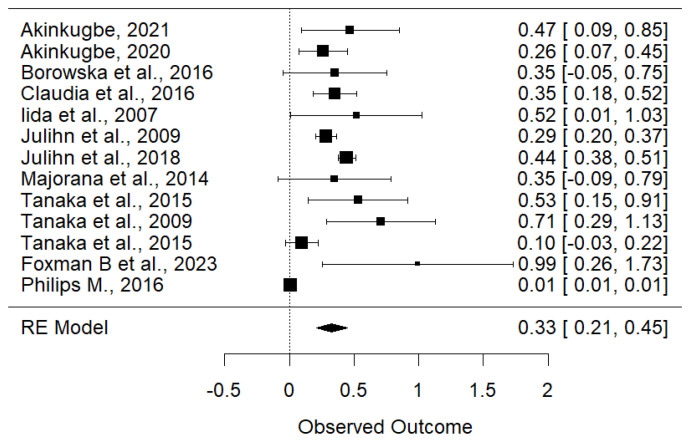
Forest plot of the association between maternal smoking during pregnancy and risk of childhood dental caries [4,16,17,18,19,20,21,22,23,24,25,26].

**Figure 5:**
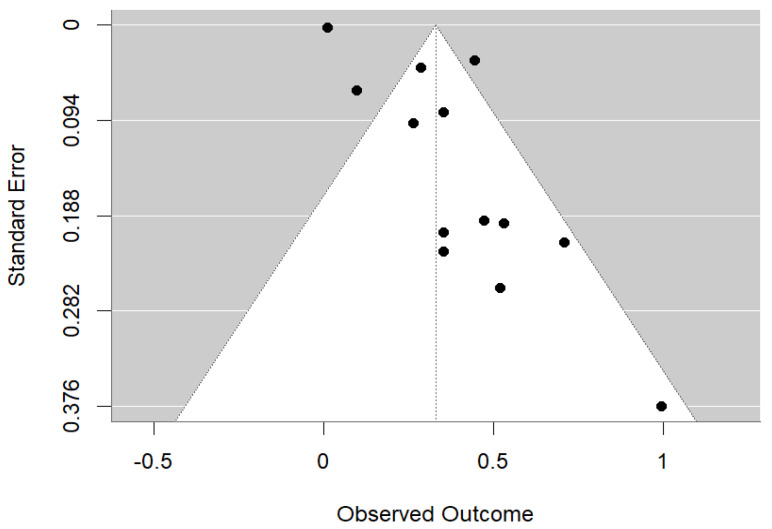
Funnel plot assessing publication bias in studies of maternal smoking and childhood dental caries.

The random-effects meta-analysis of 13 studies revealed a significant overall effect estimate (OR = 0.3290, 95% CI: 0.2089–0.4491, *p* < 0.0001) indicating a strong association between maternal smoking and childhood dental caries risk (Figure 4). However, the analysis also showed substantial heterogeneity among the studies, with an I^2^ value of 90.66%, Q test = 255.5464, *p* < 0.0001 (Figure 5), suggesting that most of the observed variability is due to differences between the studies. Overall, while there is strong evidence of a positive effect, the considerable heterogeneity highlights the need to explore potential sources of variation among the studies. The funnel plot (Figure 5), Begg’s test (*p* = 0.87), and Egger’s test (*p* = 0.51) were indicative of no publication bias for the correlation of prenatal smoking and childhood caries.

## 4. Discussion

This systematic review comprehensively explored the influence of maternal nutrition and lifestyle factors on children’s oral health. The findings indicate significant associations between maternal behaviors during pregnancy and the oral health outcomes of their offspring. These results underscore the crucial role of maternal health in the early establishment of dental health trajectories in children. Our analysis highlighted a robust link between adequate maternal intake of vitamin D and a lower incidence of enamel hypoplasia in children. Studies within our review demonstrated that children born to mothers who maintained a high level of vitamin D during pregnancy had significantly better outcomes in terms of enamel defects (enamel hypoplasia, hypomineralized second primary molars, and molar incisor hypomineralization) and dental carries. This finding aligns with earlier research suggesting that vitamin D is crucial for the mineralization of dental enamel during intrauterine development [27]. The effects of maternal smoking during pregnancy were notable, with increased risks for dental caries in children. Studies have provided contrasting results regarding the duration and timing of tobacco exposure. Akinkugbe’s study suggested that maternal smoking during the third trimester was associated with a higher risk of childhood caries [16]. However, contrasting results were reported by Tanaka et al. in a separate study, which found a stronger link between maternal smoking in the first trimester and childhood caries compared to other stages of pregnancy [4]. Nicotine exposure significantly alters the oral microbiome, creating conditions that favor the proliferation of caries-causing bacteria like *Streptococcus mutans* and *Lactobacillus* species [28]. This shift in bacterial composition has serious implications for dental health. Nicotine enhances bacterial adhesion to tooth surfaces, stimulates biofilm formation through increased production of extracellular polysaccharides, and accelerates acid production by these harmful bacteria. Additionally, it alters saliva composition, impairs local immune responses, and may provide additional nutrients for bacterial growth. These factors collectively create an environment conducive to tooth decay by promoting the formation of acidic biofilms that erode tooth enamel. The nicotine-induced disruption of the oral ecosystem’s natural balance further compounds these effects, potentially suppressing beneficial bacteria while allowing cariogenic species to thrive, thereby significantly increasing the risk of dental caries. Maternal smoking during pregnancy can harm children’s dental health by disrupting dental pulp mineralization [29]. Nicotine interferes with dental pulp stem cells’ regenerative abilities, affecting crucial cellular processes for dental development. This exposure can lead to compromised dental pulp mineralization in children, potentially impacting their long-term oral health. Nicotine also negatively impacts serum vitamin D levels through multiple pathways [30]. It reduces vitamin D synthesis by increasing oxidative stress, damages the liver’s ability to convert vitamin D to its active form, disrupts calcium balance, and alters parathyroid hormone production. Research has established a clear link between tobacco use and lower vitamin D levels, demonstrating nicotine’s harmful effect on vitamin D maintenance. Studies on smoking cessation and secondhand smoke exposure in children further illustrate nicotine’s wide-ranging influence on serum biomarkers and vitamin D metabolism. These findings highlight the complex interplay between nicotine exposure and vitamin D deficiency, emphasizing the importance of considering tobacco use in assessing and managing vitamin D status. These lifestyle factors are modifiable, and our findings reinforce the importance of targeted public health interventions aimed at promoting healthier behaviors among expectant mothers. The public health implications are profound, considering that prenatal exposure to tobacco has been consistently linked to adverse health outcomes in offspring, including oral health deficits. This systematic review and meta-analysis offers several significant advancements over previous ones [27,31,32]. Unlike earlier reviews that examined maternal cigarette smoking and vitamin D status in isolation, our study provides a comprehensive analysis of both factors and their potential interplay. By incorporating recent studies, we were able to conduct a more robust meta-analysis, which yielded statistically significant associations between both maternal vitamin D status and smoking during pregnancy with childhood caries risk and enamel defects. Notably, our findings provide stronger evidence for the link between maternal vitamin D levels and enamel defects and caries, an area where previous reviews had been inconclusive. Furthermore, this study goes beyond mere association to explore potential biological mechanisms underlying these relationships. By examining how both factors independently and synergistically affect oral health outcomes in offspring, our review not only confirms these important associations but also suggests a more holistic approach to prevention. This comprehensive perspective underscores the complex interrelationships between maternal behaviors, nutritional status, and children’s oral health, paving the way for more targeted and effective interventional strategies during pregnancy to improve long-term dental outcomes in children. In this systematic review, several potential sources of bias were identified that could influence the interpretation of our findings. The variability in methodologies used across the included studies introduces heterogeneity in the assessment of cigarette exposure, caries detection, and vitamin D status. The methodologies employed for caries detection exhibited considerable variability across the included studies. Most studies relied solely on visual examination protocols. Some studies implemented a combined tactile and visual assessment approach. Others reported parent-reported carries. Other studies extracted data from databases. Notably, only one study incorporated radiographic examination findings in their reported childhood caries data, thus providing a more comprehensive diagnostic perspective [22]. Studies also exhibited significant variability in their reporting of vitamin D status and supplementation practices. Supplementation rates fluctuated dramatically across the research, spanning from a complete absence to a substantial majority. This wide range suggests considerable heterogeneity in vitamin D supplementation approaches among the studied populations. Vitamin D levels, when reported also showed marked differences. Among the three studies that provided these data, the mean or median values ranged from a high of 43.4 ng/mL to a low median of 19.2 ng/mL [7,10,13]. Notably, only one study offered a direct comparison between high-dose and standard-dose supplementation groups, revealing mean Vitamin D levels of 43.4 ng/mL and 28.9 ng/mL, respectively [7]. These factors collectively contributed to potential biases that must be considered in evaluating the overall evidence and its implications.

## 5. Limitations and Future Research

While our review provides comprehensive insights, there are limitations. The heterogeneity in study designs, measurements, and populations across included studies may hinder the generalizability of our findings. The predominance of cross-sectional study designs prevents establishing a clear causal relationship between maternal smoking and childhood caries. Additionally, many of the included studies did not adequately control key confounding factors, such as passive smoking, which could have influenced the overall results. The review was also constrained by the lack of data on caries severity levels, limiting the ability to assess the nuanced impacts of maternal smoking. Finally, the heavy reliance on self-reported exposure data introduces the possibility of response bias, potentially affecting the validity of the meta-analysis findings. This variability also underscores the complexity of how different factors influence oral health and suggests that additional factors such as genetic predispositions and socio-economic status need to be considered in future research.

## 6. Conclusions

The evidence from this systematic review clearly indicates that maternal vitamin D intake and tobacco smoking have a direct impact on the oral health of children. The analysis found a significant overall association between adequate maternal vitamin D intake and a reduced risk of enamel defects, such as enamel hypoplasia, hypomineralized second primary molars, and molar incisor hypomineralization, in children. However, the analysis also revealed substantial heterogeneity among the studies, likely due to differences in study designs, populations, and assessment methods. Our review also consistently demonstrated a strong association between maternal smoking during pregnancy and an increased risk of early childhood dental caries in children. The potential mechanisms include nicotine-induced disruption of the oral microbiome, promotion of cariogenic bacteria, impairment of dental pulp mineralization, and interference with vitamin D metabolism. These findings underscore the critical importance of maternal health behaviors, particularly vitamin D status and smoking cessation, in shaping the early oral health trajectories of children. Public health interventions targeting these modifiable factors during pregnancy could have significant implications for improving children’s dental health outcomes. Most importantly, this review highlights the necessity for integrative approaches that include dietary guidance, cessation of smoking during pregnancy, and the promotion of early dental hygiene practices. By addressing these factors, healthcare providers can contribute to the foundational health of children, starting with pregnancy. Our findings should serve as a call to action for policymakers to consider oral health as an integral part of prenatal and postnatal care strategies, potentially leading to substantial improvements in public health outcomes.

## Figures and Tables

**Table 1:** Summary of study characteristics and outcomes included in the assessment of the impact of maternal vitamin D on dental defects in children.

Author, Year	Country	Study Design	Sample Size	Outcomes
Mortensen et al., 2022 [10]	Denmark	Retrospective Cohort	1241	Prevalence of HSPM: 54.7%. No significant association between 25(OH) D and HSPM.
Beckett et al., 2022 [8]	New Zealand	Retrospective Cohort	81	Vitamin D insufficiency (<50 nmol/L) in the third trimester was associated with an increased risk of caries by age 6 (IRR: 3.55, CI: 1.15–10.92).
Børsting et al., 2022 [11]	Norway	Randomized Trial	176 pairs	Prevalence: 32% MIH, 22% HSPM. Insufficient maternal vitamin D during gestational weeks 18–22 was significantly associated with MIH (adjusted RR: 1.82, 95% CI: 1.13–2.93).
Nørrisgaard et al., 2019 [7]	Denmark	Randomized Trial	623 mothers; 588 of their children	High-dose vitamin D (2400 IU/day) supplementation during pregnancy was associated with a lower risk of enamel defects in permanent OR: 0.47 (95% CI: 0.27–0.81) and deciduous dentitions OR: 0.50 (95% CI: 0.28–0.87) of the offspring.
Van der Tas et al., 2018 [9]	The Netherland	Prospective Cohort	4750 mothers; 3983 children	No significant association between 25(OH) D concentrations in prenatal, early postnatal, and later postnatal life and the presence of HSPMs or MIH at the age of six.
Reed et al., 2017 [12]	USA	Randomized Trial	29 Pairs	EH prevalence: 45%. Maternal vitamin D insufficiency might be associated with EH in children’s teeth.
Schroth et al., 2014 [13]	USA	Prospective Cohort	207	Mothers of children with ECC had significantly lower 25(OH) D levels compared to those whose children were caries-free (41 ± 20 nmol/L vs. 52 ± 27 nmol/L; *p* = 0.05).
M. J. Silva et al., 2018 [14]	Australia	Prospective Cohort	250 twin pregnancies; 344 twins	Prevalence of HSPM was 19.8%. No significant evidence (*p* = 0.172) was found for genetic influence on HSPM. Maternal smoking in the second OR: 2.84, 95% CI (1.28–6.30), *p* = 0.01 and third OR: 3.23, 95% CI (1.42–7.33), *p* = 0.05 trimester was associated with HSPM.
Neto et al., 2020 [15]	Brazil	Cross-section	152	Children with vitamin D deficiency during gestation were 6.40 times more likely to have DDE.

HSPM: Hypomineralized second primary molar; MIH: Molar incisor hypomineralization; ECC: Early childhood carries; DDE: Developmental defect of enamel; EH: Enamel hypoplasia; 25(OH) D: Serum 25-hydroxyvitamin D; OR: Odds ration; RR: Relative risk.

**Table 2:** Summary of the characteristics of this study included in the assessment of the effect of maternal smoking on childhood carries.

Author, Year	Country	Study Design	Sample Size	Outcomes
Akinkugbe, 2021 [16]	England	Retrospective Cohort	1429	RR: 1.60 (1.09–2.32)
Akinkugbe, 2020 [17]	England	Prospective Cohort	1429	RR: 1.30 (1.08–1.58)
Borowska-Struginska et al., 2016 [18]	Poland	Cross-section	1131	OR: 1.42 (0.95–2.12)
Claudia et al., 2016 [19]	Australia	Retrospective Cohort	1687	RR: 1.42 (1.20–1.68)
Iida et al., 2007 [20]	USA	Cross-section	1576	OR: 1.68 (1.01–2.79)
Julihn et al., 2009 [21]	Sweden	Cross-section	15,538	OR: 1.33 (1.22–1.44)
Julihn et al., 2018 [22]	Sweden	Cross-section	65,259	OR: 1.56 (1.42–1.63)
Majorana et al., 2014 [23]	Italy	Cross-section	2395	OR: 1.42 (0.92–2.21)
Tanaka et al., 2015 [24]	Japan	Cross-section	6412	OR: 1.70 (1.15–2.48)
Tanaka et al., 2009 [4]	Japan	Cross-section	2015	OR: 2.03 (1.33–3.09)
Tanaka et al., 2015 [24]	Japan	Retrospective Cohort	76,920	HR: 1.10 (0.97–1.25)
Foxman B et al., 2023 [25]	USA	Prospective Cohort	1015	OR: 2.7 (1.29–5.64)
Philips M., 2016 [26]	USA	Retrospective Cohort	917	OR: 1.01 (1.01–1.02)

OR: Odds ration; RR: Relative risk; HR: Hazard ratio.

## Data Availability

All data analyzed during this study are included in this article.

## Supplementary Materials

The following supporting information can be downloaded at: https://www.mdpi.com/article/10.3390/children11091107/s1. Table S1: Summary of search strategy for PubMed; Table S2: Summary of search strategy for Scopus; Table S3: Summary of search strategy for Web of Science.
