# Supplementary material for: Influences of Maternal Nutrition and Lifestyle Factors on Early Childhood Oral Health: A Systematic Review of Mechanisms and Intervention Strategies
Source: Children (Basel). 2024 Sep 10;11(9):1107. doi: 10.3390/children11091107 (PMC11430575; doi:10.3390/children11091107)
Supplement: Supplementary file 1 [file children-11-01107-s001.zip › children-3157846-supplementary.pdf]

children MDPI

Table S1: Summary of search strategy for PubMed

Step	Search Terms	Search Field	Boolean Operator	Search Filters	Results
1	"Maternal Nutrition"	Title/Abstract		Date: 2000-2024, English	2273
2	"Maternal Nutritional physiological phenomena" [MeSH]	MeSH	OR	Date: 2000-2024, English	7852
3	"Maternal Vitamin D intake"	Title/Abstract	OR	Date: 2000-2024, English	7878
4	"Maternal Calcium intake"	Title/Abstract	OR	Date: 2000-2024, English	7897
5	"Maternal Factors"	Title/Abstract	OR	Date: 2000-2024, English	10457
6	"Smoking" [MeSH] AND "Pregnancy" [MeSH]	MeSH	OR	Date: 2000-2024, English	14044
7	"Obesity" [MeSH] AND "Pregnancy" [MeSH]	MeSH	OR	Date: 2000-2024, English	23478
8	"Maternal Alcohol Intake"	Title/Abstract	OR	Date: 2000-2024, English	23528
9	"Maternal Lifestyle"	Title/Abstract	OR	Date: 2000-2024, English	23739
10	1 OR 2 OR 3 OR 4 OR 5 OR 6 OR 7 OR 8 OR 9	-	AND	Date: 2000-2024, English	
11	"Oral Health"	Title/Abstract		Date: 2000-2024, English	61
12	"Dental Caries"	Title/Abstract	OR	Date: 2000-2024, English	27724
13	"Early Childhood Oral Health"	Title/Abstract	OR	Date: 2000-2024, English	27823
14	"Pediatric Dental Health"	Title/Abstract	OR	Date: 2000-2024, English	27828
15	"Early Childhood Caries"	Title/Abstract	OR	Date: 2000-2024, English	28244
16	"Enamel Hypoplasia"	Title/Abstract	OR	Date: 2000-2024, English	29566
17	11 OR 12 OR 13 OR 14 OR 15 OR 16	-	AND	Date: 2000-2024, English	
18	10 AND 17	-		Date: 2000-2024, English	84

Table S2: Summary of search strategy for Scopus

Step	Search Terms	Search Field	Boolean Operator	Search Filters	Results
1	"Maternal Nutrition"	Title/Abstract/Keywords		Year: 2000-2024, English	2658
2	"Maternal Vitamin D intake"	Title/Abstract/Keywords	OR	Year: 2000-2024, English	27459
3	"Maternal Calcium intake"	Title/Abstract/Keywords	OR	Year: 2000-2024, English	27923
4	"Maternal Factors"	Title/Abstract/Keywords	OR	Year: 2000-2024, English	28750
5	"Maternal Smoking"	Title/Abstract/Keywords	OR	Year: 2000-2024, English	29874
6	"Maternal Obesity"	Title/Abstract/Keywords	OR	Year: 2000-2024, English	31298
7	"Maternal Alcohol Intake"	Title/Abstract/Keywords	OR	Year: 2000-2024, English	32764
8	"Maternal Lifestyle"	Title/Abstract/Keywords	OR	Year: 2000-2024, English	33679
9	1 OR 2 OR 3 OR 4 OR 5 OR 6 OR 7 OR 8	-	AND	Year: 2000-2024, English	
10	"Oral Health"	Title/Abstract/Keywords		Year: 2000-2024, English	82
11	"Dental Caries"	Title/Abstract/Keywords	OR	Year: 2000-2024, English	32387
12	"Early Childhood Oral Health"	Title/Abstract/Keywords	OR	Year: 2000-2024, English	34867
13	"Pediatric Dental Health"	Title/Abstract/Keywords	OR	Year: 2000-2024, English	34765
14	"Early Childhood Caries"	Title/Abstract/Keywords	OR	Year: 2000-2024, English	35476
15	"Enamel Hypoplasia"	Title/Abstract/Keywords	OR	Year: 2000-2024, English	36732
16	10 OR 11 OR 12 OR 13 OR 14 OR 15	-	AND	Year: 2000-2024, English	
17	9 AND 16	-		Year: 2000-2024, English	94

Table S3: Summary of search strategy for Web of science

Step	Search Terms	Search Field	Boolean Operator	Search Filters	Results
1	“Maternal Nutrition”	Topic		Year: 2000-2024, English	2129
2	“Maternal Vitamin D intake”	Topic	OR	Year: 2000-2024, English	17459
3	“Maternal Calcium intake”	Topic	OR	Year: 2000-2024, English	17924
4	“Maternal Factors”	Topic	OR	Year: 2000-2024, English	18750
5	“Maternal Smoking”	Topic	OR	Year: 2000-2024, English	18771
6	“Maternal Obesity”	Topic	OR	Year: 2000-2024, English	19298
7	“Maternal Alcohol Intake”	Topic	OR	Year: 2000-2024, English	19764
8	“Maternal Lifestyle”	Topic	OR	Year: 2000-2024, English	19972
9	1 OR 2 OR 3 OR 4 OR 5 OR 6 OR 7 OR 8		AND	Year: 2000-2024, English	
10	“Oral Health”	Topic		Year: 2000-2024, English	74
11	“Dental Caries”	Topic	OR	Year: 2000-2024, English	17385
12	“Early Childhood Oral Health”	Topic	OR	Year: 2000-2024, English	18863
13	“Pediatric Dental Health”	Topic	OR	Year: 2000-2024, English	19765
14	“Early Childhood Caries”	Topic	OR	Year: 2000-2024, English	20475
15	“Enamel Hypoplasia”	Topic	OR	Year: 2000-2024, English	21732
16	10 OR 11 OR 12 OR 13 OR 14 OR 15	-	AND	Year: 2000-2024, English	
17	9 AND 16	-		Year: 2000-2024, English	60
